# Barriers to Patient Safety in Ambulatory Surgical Centers: Lessons Learned from an Outbreak of Mycobacterium fortuitum Joint Infections

**DOI:** 10.1017/ash.2025.234

**Published:** 2025-09-24

**Authors:** Simone Godwin, Becky Meyer, Kelley Tobey, Tracey Rhodes, Corinne Tandy, Edwards Megan, Christopher Wilson

**Affiliations:** 1 Tennessee Department of Health; 2 TN Dept of Health; 3 TN Department of Health; 4 Knox County Health Department

## Abstract

**Background:** Procedures performed at Ambulatory Surgical Centers (ASCs) have been increasing in type and volume for over a decade. Similarly, outbreaks in ASCs are increasingly detected, but ASCs face unique challenges to Infection Prevention and Control (IPC). In 2023, Tennessee state and local health departments (HDs) responded to an outbreak of 14 Nontuberculous mycobacteria (NTM) periprosthetic joint infections in an ASC, unveiling gaps in IPC practice and significant barriers to resolving them. **Method:** Cases were detected through third-party clinical laboratory reporting. HD Infection Preventionists (IPs) conducted on-site infection control assessments using qualitative observation, verbal interview, and CDC’s Infection Control Assessment and Response (ICAR) and Association of perioOperative Registered Nurses (AORN) checklist tools. A citizen complaint triggered an independent survey performed by the state’s regulatory body. **Result:** ICAR revealed there was no Water Management Plan (WMP) for the building or ASC suite. Areas with lapses in IPC practice included aseptic technique, instrument handling, and environmental services (EVS). There was no surveillance mechanism for tracking surgical site infections. Complications were tracked via paper provider surveys but could not be produced when requested. Regulatory survey identified additional violations related to biohazardous waste and unlicensed performing of pediatric procedures.

The facility IP and the sterile processing department lacked specialized training in their respective areas. The IP had no knowledge of reportable disease requirements. The outbreak was reported by the clinical laboratory only after five cases had been detected at a separate facility where revisions were performed. **Conclusion:** Major barriers to IPC best practice included lack of subject matter expertise and the complexity of multi-stakeholder ownership and operation. A healthcare management corporation holding the facility license was responsible for ASC operations, employment of non-physician staff, and adherence to state and federal regulations. An independent orthopedic group employed surgeons, and a third healthcare system owned the building and contracted EVS. As a result, the licensee was not capable of addressing building water management, and the facility IP had no authority over EVS or the physicians’ group to require complications reporting. Public health action was delayed by the ASC not reporting the outbreak, despite NTM being reportable in Tennessee. This delay was likely due to lack of knowledge around reportable diseases and poor surveillance and follow-up. Once all stakeholders met, compliance with recommended interventions improved. Public health authorities should consider supporting ASC IP education opportunities, engaging varied stakeholders during outbreaks, and enhancing surveillance within this setting.

